# Blockade of Na_V_1.8 Increases the Susceptibility to Ventricular Arrhythmias During Acute Myocardial Infarction

**DOI:** 10.3389/fcvm.2021.708279

**Published:** 2021-08-02

**Authors:** Baozhen Qi, Shimo Dai, Yu Song, Dongli Shen, Fuhai Li, Lanfang Wei, Chunyu Zhang, Zhenning Nie, Jiaxiong Lin, Lidong Cai, Junbo Ge

**Affiliations:** ^1^Department of Cardiology, Zhongshan Hospital, Shanghai Institute of Cardiovascular Disease, Fudan University, Shanghai, China; ^2^Department of Cardiology, Shanghai General Hospital, School of Medicine, Shanghai Jiao Tong University, Shanghai, China

**Keywords:** SCN10A, cardiac ganglionated plexi, ventricular arrhythmia, acute myocardial infarction, sodium channel

## Abstract

*SCN10A*/Na_V_1.8 may be associated with a lower risk of ventricular fibrillation in the setting of acute myocardial infarction (AMI), but if and by which mechanism Na_V_1.8 impacts on ventricular electrophysiology is still a matter of debate. The purpose of this study was to elucidate the contribution of Na_V_1.8 in ganglionated plexi (GP) to ventricular arrhythmias in the AMI model. Twenty beagles were randomized to either the A-803467 group (*n* = 10) or the control group (*n* = 10). Na_V_1.8 blocker (A-803467, 1 μmol/0.5 mL per GP) or DMSO (0.5 mL per GP) was injected into four major GPs. Ventricular effective refractory period, APD_90_, ventricular fibrillation threshold, and the incidence of ventricular arrhythmias were measured 1 h after left anterior descending coronary artery occlusion. A-803467 significantly shortened ventricular effective refractory period, APD_90_, and ventricular fibrillation threshold compared to control. In the A-803467 group, the incidence of ventricular arrhythmias was significantly higher compared to control. A-803467 suppressed the slowing of heart rate response to high-frequency electrical stimulation of the anterior right GP, suggesting that A-803467 could inhibit GP activity. *SCN10A*/Na_V_1.8 was readily detected in GPs, but was not validated in ventricles by quantitative RT-PCR, western blot and immunohistochemistry. While *SCN10A*/Na_V_1.8 is detectible in canine GPs but not in ventricles, blockade of Na_V_1.8 in GP increases the incidence of ventricular arrhythmias in AMI hearts. Our study shows for the first time an influence of *SCN10A*/Na_V_1.8 on the regulation of ventricular arrhythmogenesis via modulating GP activity in the AMI model.

## Introduction

Acute myocardial infarction (AMI) always results in electrical instability and fatal ventricular arrhythmias which leads to sudden cardiac death. Ventricular fibrillation (VF) is one of the most common acute ventricular arrhythmias during AMI, which is associated with autonomic imbalance (1). The cardiac autonomic nervous system involves of both the intrinsic and extrinsic components. The intrinsic cardiac autonomic nervous system (ICANS) is a complex neural network formed by interconnecting nerves and ganglionated plexi (GP) concentrated in epicardial fat pads (2).

*SCN10A* encodes the alpha subunit of Na_V_1.8. Na_V_1.8 is a tetrodotoxin-resistant sodium channel and most highly expressed in dorsal root ganglia, which is reported to function in the transmission of pain signals (3). Recently, Na_V_1.8 has been identified in intrinsic cardiac nerve fibers and the cardiac GP (4–6). Several studies have shown that *SCN10A*/Na_V_1.8 is associated with myocardial repolarization and cardiac conduction, as well as atrial fibrillation (AF) and VF (7, 8). Data from Chambers et al. indicated that a common variant in *SCN10A* is associated with a lower risk of VF during AMI (7). However, if and by which mechanism *SCN10A*/Na_V_1.8 impacts on cardiac electrophysiology is still a matter of debate.

Inhibition of Na_V_1.8 by the blocker A-803467 has been reported to decrease late *I*_*Na*_ and shorten APD in mouse and rabbit cardiomyocytes (9), whereas the absence of functional Na_V_1.8 has been described in non-diseased atrial and ventricular cardiomyocytes (6, 10). We and others have shown that Na_V_1.8 plays a critical role in cardiac conduction via modulation of AP firing in intracardiac neurons (4, 6, 11). In the present study, we investigated the functional relevance of Na_V_1.8 in cardiac GP, focusing on the contribution of Na_V_1.8 to ventricular electrophysiology and ventricular arrhythmias in the AMI model.

## Materials and Methods

### Surgical Preparation and Experimental Design

The animal protocol used for this study was reviewed and approved by the Institutional Animal Care and Use Committee of Zhongshan Hospital, Fudan University, and conformed to the Guide for the Care and Use of Laboratory Animals by the US National Institutes of Health (Publication No. 85-23, revised 1996). Twenty male beagles weighting 12 to 15 kg were anesthetized by administrating an intramuscular injection of xylazine (2.2 mg/kg) and ketamine (30 mg/kg), and maintained by 1–2% isoflurane/O_2_. All measures were taken to minimize suffering. All animals were euthanized with a lethal dose of pentobarbital at the end of the experiments (100 mg/Kg, IV).

**Figure 1:**
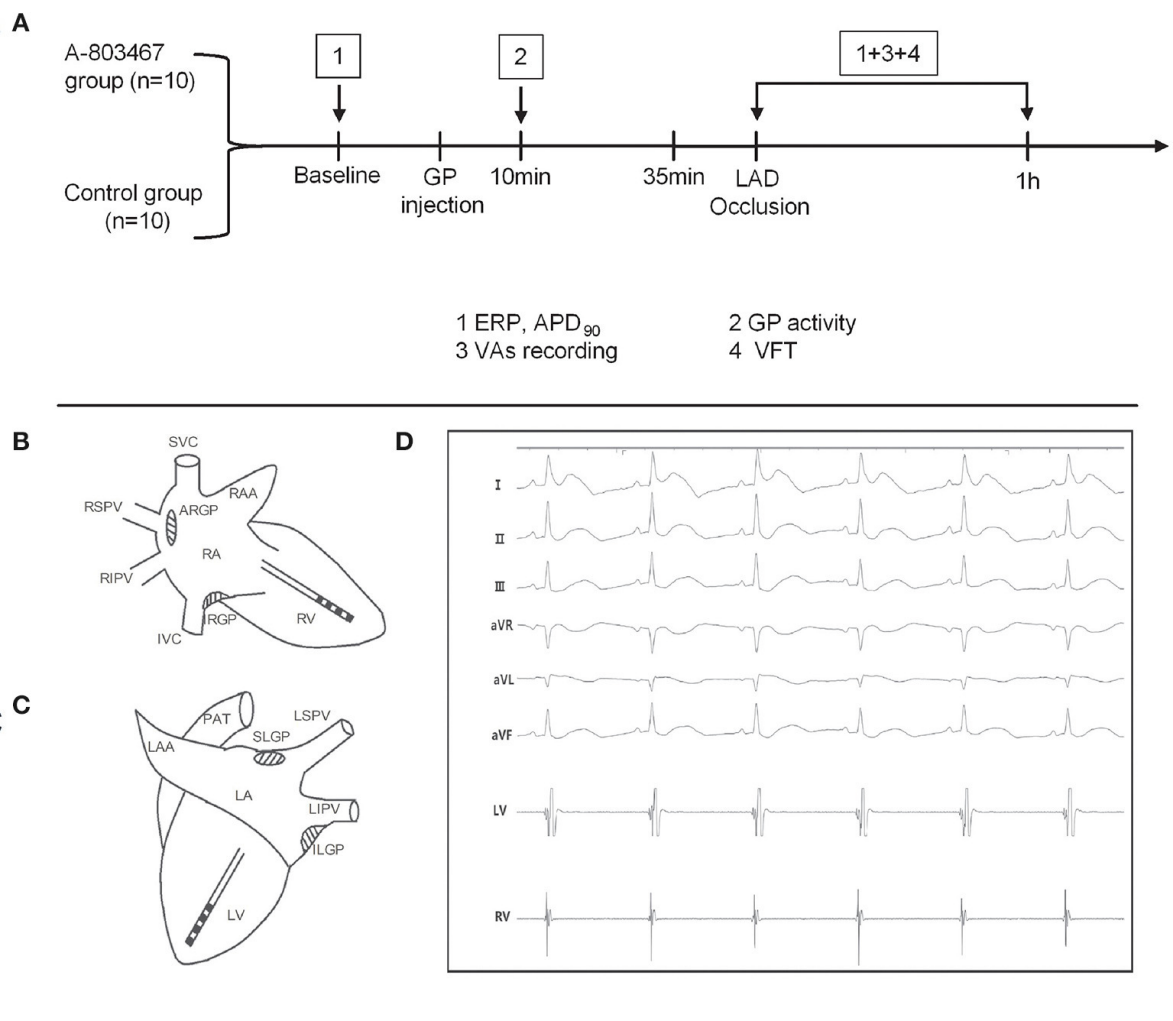
Schematic representation of the study protocol **(A)** and catheter positions in the right **(B)** and left **(C)** ventricular free walls. **(D)** Surface electrocardiograms and local cardiac electrograms recording after left anterior descending coronary artery occlusion.

Twenty beagles were randomized to either the A-803467 group (*n* = 10) or the control group (*n* = 10). A-803467 (TargetMol, T2024) was dissolved in DMSO. The study protocol is shown in Figure 1A. Both right and left thoracotomies were performed at the fourth intercostal space, and the pericardium was opened. A-803467 (1 μmol/0.5 mL at each GP) or DMSO (0.5 mL at each GP, control) was injected into four major GPs within four epicardial fat pads: the superior left GP, the anterior right GP (ARGP), the inferior left GP, and the inferior right GP (Figures 1B,C). High frequency electrical stimulation (HFS, 0.1 ms duration, 20 Hz, square waves) was applied by a bipolar electrode probe (AtriCure, West Chester, Ohio, USA) to identify the cardiac GP. And the response was a progressive slowing of heart rate (HR) or atrioventricular conduction.

### Electrophysiological Study Protocol

Two multi-electrode catheters (Capsure Epi, Medtronic, Minneapolis, MN, USA) were sutured on the left and right ventricular free walls to perform ventricular pacing with twice the diastolic pacing threshold (Figures 1B,C). Electrocardiographic and intracardiac electrograms were recorded on a Bard Computerized Electrophysiology system (Figure 1D, CR Bard Inc., Bard, Billerica, Massachusetts, USA) and were filtered and amplified from 0.05 to 500 Hz.

Ventricular pacing was performed at a cycle length of 300 ms (S1-S1). Ventricular effective refractory period (VERP) was started at 250 ms and repeated with progressively shorter S1-S2 intervals until the ventricular capture failed (S1:S2 = 8:1). VERP was defined as the longest coupling interval that did not capture the ventricle.

The monophasic action potential (MAP) was recorded by a catheter (Foehr Medical Instruments GMBH, Seeheim, Germany) at the left ventricle during atrial pacing with a custom-made Ag-AgCl electrode sutured on the right atrial appendage (BCL = 340 ms). The 90% of action potential duration (APD_90_) was defined as MAP measured at 90% repolarization.

In order to verify whether the action of A-803467 was mediated by regulating GP function, we examined GP activity at 10 min after A-803467 or DMSO injection into the ARGP. HFS (square waves, 0.1 ms duration, 20 Hz) was applied to the ARGP with increasing voltages, and voltage-HR response curves were constructed. Changes of HR response to ARGP stimulation at different voltages were used as the surrogate marker for GP activity (12).

### AMI Protocol and Ventricular Arrhythmias

As the largest action of A-803467 was observed at 35 min after local injection (13), which lasted at least 90 min, we occluded left anterior descending coronary artery (LAD) at 35 min after drug injection. LAD was occluded by using 3.0 silk suture positioned at approximately half of the distance from the apex. Then the incidence of ventricular arrhythmias including ventricular tachycardia (VT), ventricular premature contraction (VPC), and VF were recorded during 1 h after LAD occlusion.

Ventricular fibrillation threshold (VFT) was determined with right ventricular pacing at 1 h after LAD occlusion. S1-S1 (100 ms) was applied at the end of a 20-beat drive train with a pacing cycle length of 300ms. VFT was determined by progressively increase of pacing current in 2V steps. The minimum voltage required producing sustained VF was defined as VFT. Hearts were defibrillated with direct-current cardioversion.

### Quantitative Real-time Polymerase Chain Reaction

Cardiac GPs, atria and ventricles were snap frozen in liquid N_2_, and stored at −80°C until ready for quantitative real-time polymerase chain reaction (qPCR) and western blot analysis. Total RNA was isolated with RNeasy Mini Kit (74104, Qiagen) and was digested with DNase I (79254, Qiagen) according to manufacturer's instructions. cDNA was synthesized using the iScript cDNA Synthesis Kit (170-8891, Bio-Rad). Sequences of the primers are as follows: *SCN10A*, forward, 5′- CACCAGCTTTGATTCCTTTGC-3′ and reverse: 5′-ATTTTCCCAGATGCCCTCAG-3′ and β*-actin*, forward, 5′-TCCACGAGACCACTTTCAAC-3′ and reverse: 5′-TTTCCTTCTGCATCCTGTCG-3′. qPCR was performed on a 7500 Fast Real-Time PCR system (ABI, UK) with the SYBR Green PCR kit (Takara), and β-actin was used as an internal control. Each sample was analyzed in triplicate.

### Western Blot Analysis

Samples were lysed with RIPA buffer (Beyotime, China) and the concentration of protein was assayed using a BCA Protein Assay Kit (Sigma-Aldrich, St. Louis, MO, USA). A total of 10 μg of protein was separated on SDS-PAGE (10 %) at 80 V for 1.5 h, and transferred onto PVDF membranes (Bio-Rad, USA) at 300 mA for 1.5 h. The following primary antibodies: Na_V_1.8 (1:500, ab114110, Abcam) and β-actin (1:5000, ab8227, Abcam, Cambridge, UK) were used to incubate the blots at 37 °C for 2 h with gentle shaking. Afterwards, the secondary antibody Goat Anti-Rabbit IgG H&L (HRP) (1:5000; ab205718, Abcam, Cambridge, UK) was used to incubate the blots for 1 h at room temperature. The band density was analyzed using a gel imaging system and compared with an internal control.

### Immunohistochemistry

Cardiac GPs, atria and ventricles were fixed with 10% formalin and embedded in paraffin. Then, 5-μm-thick tissues were serially cut from paraffin blocks and mounted onto glass slides coated with 1% (W/V) gelatin solution. After antigen retrieval, the tissue sections were incubated with primary antibodies against Na_V_1.8 α subunit (1:20, ab114110, Abcam, Cambridge, UK) at 4°C overnight. After washing three times with PBS, the sections were then incubated with the secondary antibody (HRP labeled goat anti rabbit, 1:200, ab205718, Abcam, Cambridge, UK) expression were detected using DAB (brown) staining.

### Ischemic Size Determination

The left auricular appendage was cannulated. Evans blue solution (200 mL, 2% in physiological saline) was infused into the left atrium, left ventricle, aorta and coronary artery, which resulted in a dark blue staining of the non-ischemic area. The heart was rapidly excised, and the atria and right ventricular free wall was removed. Both the non-trained ischemic areas and the blue-stained normal areas of the left ventricular free wall and of the septum were weighed separately. The mass of the ischemic tissue was expressed as fraction of the left, or septal ventricular tissue mass.

### Statistical Analysis

Data are shown as mean ± SEM. The repeated measures analysis of variance (ANOVA) was used to compare the mean of VERP, APD_90_, and the slowing of HR with increasing voltage between the A-803467 group and the control group. The Mann-Whitney U test was used to compare the maximal percent change of HR. The independent-samples *t*-test was used to compare the mean of VPC, the duration of VT, VFT, and infarct size between two groups. Incidence of VF was compared between the A-803467 group and the control group using Fisher's exact test. GraphPad Prism software version 6.0 (GraphPad Software, La Jolla, California) was used for statistical analysis, and *P* < 0.05 was considered statistically significant.

## Results

### Effect of Blocking Na_V_1.8 on Ventricular Electrophysiology

**Figure 2:**
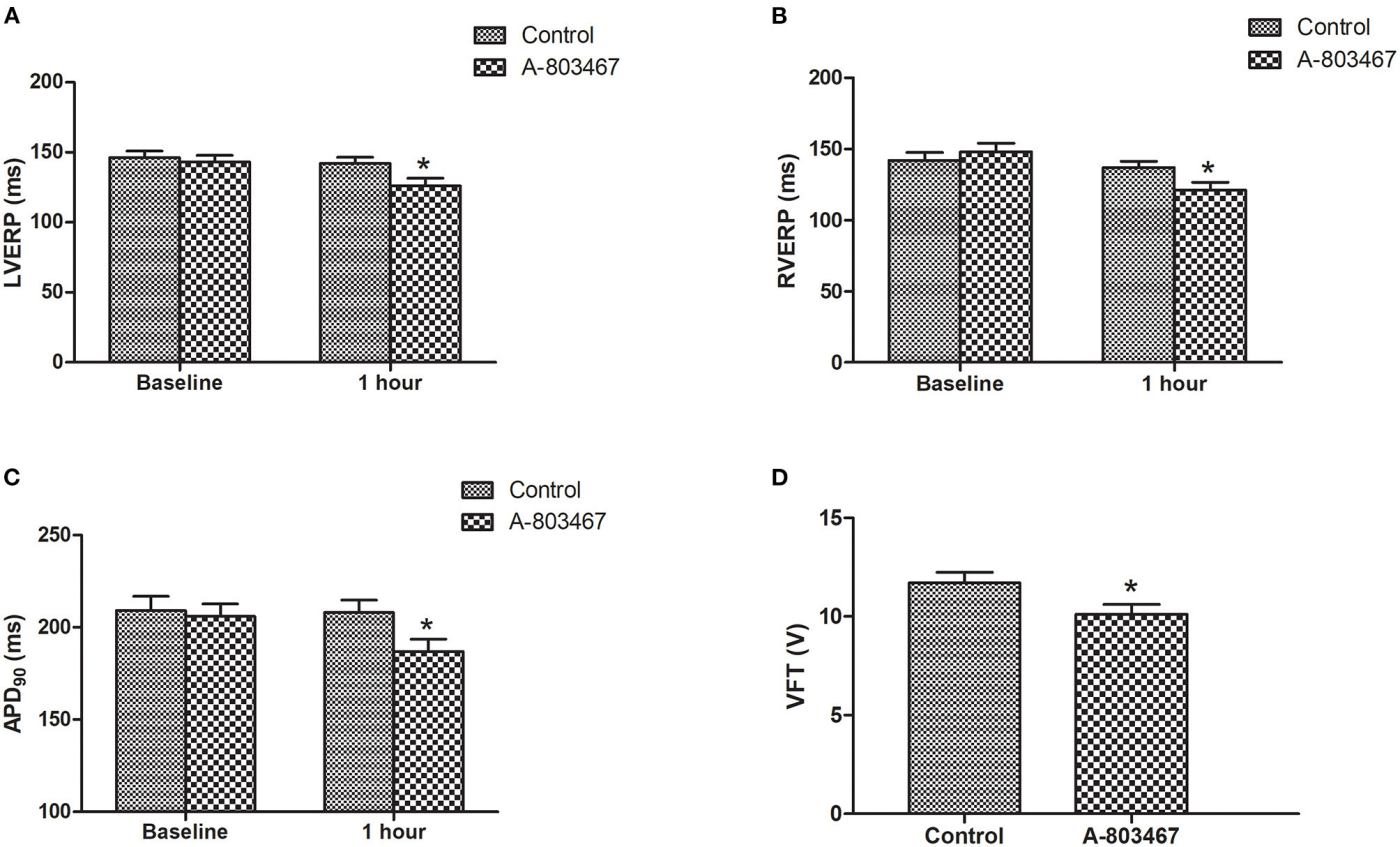
Effects of A-803467 on Ventricular electrophysiology in the both two groups. **(A)** The left ventricular effective refractory period (LVERP) was decreased in the A-803467 group compared to control. **(B)** The right ventricular effective refractory period (RVERP) was decreased in the A-803467 group compared to control. **(C)** A-803467 significantly shortened 90% of action potential duration (APD_90_) at 1 h after LAD occlusion. **(D)** The ventricular fibrillation threshold (VFT) was significantly shorter in the A-80467 group compared to control. *P < 0.05 vs. Control group.

Change of repolarization and refractoriness contributes greatly to proarrhythmic substrate. Both left ventricular effective refractory period (LVERP) and right ventricular effective refractory period (RVERP) were significantly shorter in the A-803467 group compared to control at 1 h after LAD occlusion (Figures 2A,B). A-803467 injection in cardiac GP significantly shortened ventricular APD_90_ at 1 h after LAD occlusion compared to control (*P* < 0.05, Figure 2C). For comparison between groups, VFTs were significantly shorter in the A-803467 group compared to control (*P* < 0.05, Figure 2D). A-803467 injection in cardiac GP shortened VERP, ventricular APD_90_, and decreased VFT in AMI hearts, demonstrating that blocking Na_V_1.8 in GP distant from the ventricles can influence ventricular electrophysiological properties.

### Effect of Blocking Na_V_1.8 on Ventricular Arrhythmias

**Figure 3:**
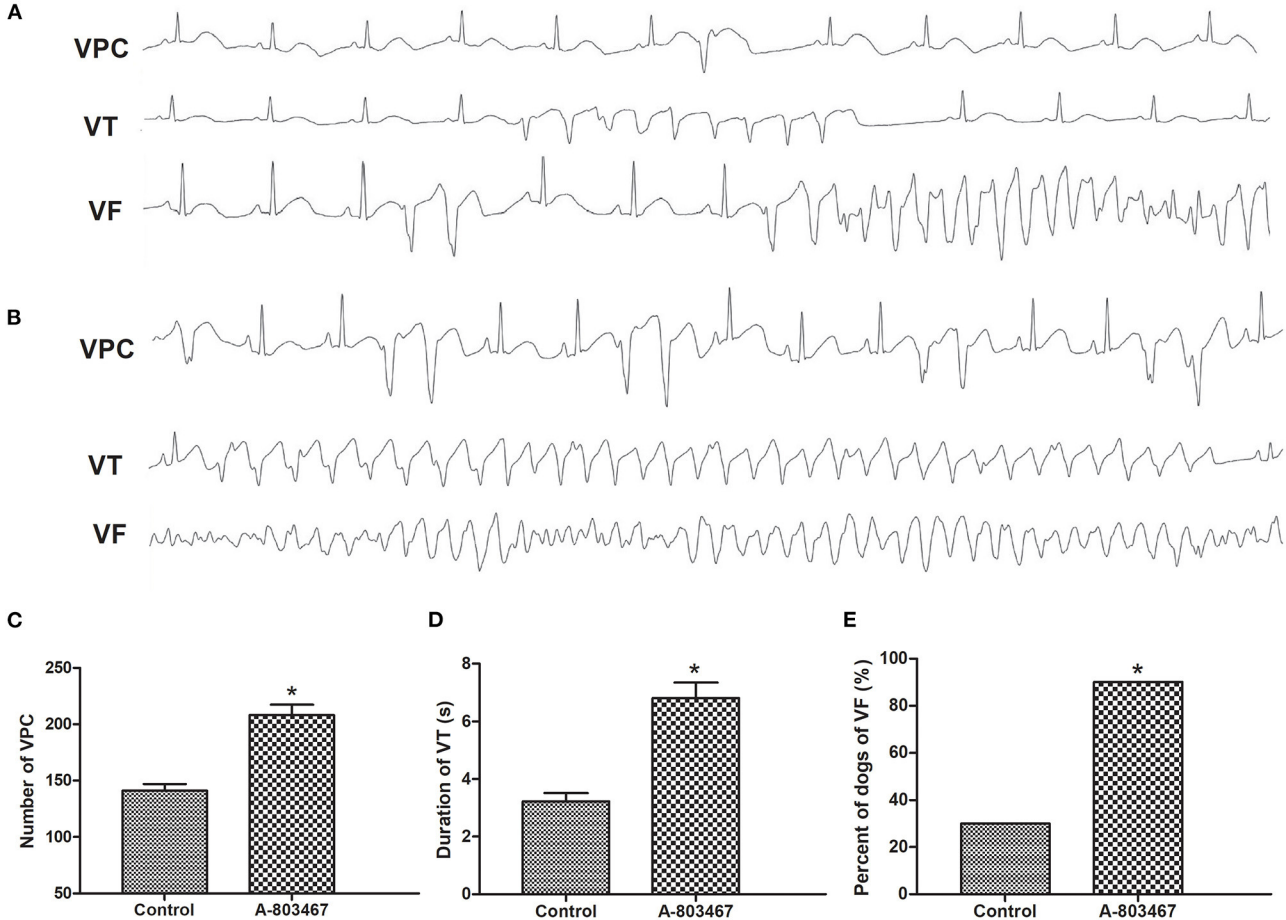
The occurrence of ventricular arrhythmias during 1 h after LAD occlusion. Representative examples of the ventricular premature contraction (VPC), ventricular tachycardia (VT), and ventricular fibrillation (VF) ECG tracings (lead II) in the Control group **(A)** and the A-803467 group **(B)**. **(C)** VPCs were markedly increased in the A-803467 group after LAD occlusion compared to Control. **(D)** The mean duration of VT was longer in the A-803467 group after LAD occlusion compared to Control. **(E)** Percentage of dogs with VF was apparently increased in the A-803467 group after LAD occlusion compared to Control. *P < 0.05 vs. Control group.

An increase in susceptibility to ventricular arrhythmia was observed during 1 h after LAD occlusion in the A-803467 group compared to control. Examples of VF, VT, and VPCs ECG tracings (lead II) were shown in Figures 3A,B. As shown in Figure 3C, the number of VPC was significantly increased in the A-803467 group compared to control (*P* < 0.05). The mean duration of VT in the A-803467 group was longer compared to control (*P* < 0.05, Figure 3D). VF occurred in only 30% (3/10) of dogs in the control group, but 90% (9/10) of dogs in the A-803467 group experienced VF during AMI (*P* < 0.05, Figure 3E).

### Effect of Blocking Na_V_1.8 on GP Activity

**Figure 4:**
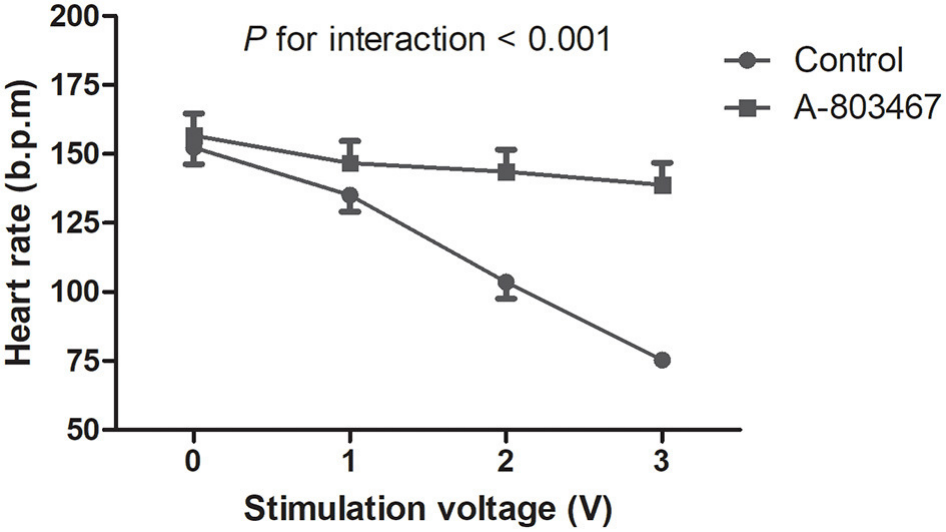
Effects of A-803467 on the ganglionated plexi activity at 10 min after local injection into the anterior right ganglionated plexi. The trend in the heart rate change with increasing stimulation voltage was significantly different between the A-803467 group and the control group.

A progressive slowing of HR was induced by HFS to the ARGP under incremental voltage levels. The maximal percent change in HR was decreased by 50.1 ± 1.3% compared to baseline in the control group, which was significantly higher compared with the A-803467 group (*P* < 0.05). HR decreased linearly with incremental stimulation voltage in the control group, whereas HR remained relatively flat after A-803467 injection. The treatment group by voltage interaction was significant, demonstrating that HR change with incremental voltage is different between the A-803467 and control group (*P* < 0.001, Figure 4). The results suggested that A-803467 could inhibit the activation of the neural elements within the GP.

### Presence of scn10a/Na_V_1.8 in Cardiac GPs and the Myocardium

**Figure 5:**
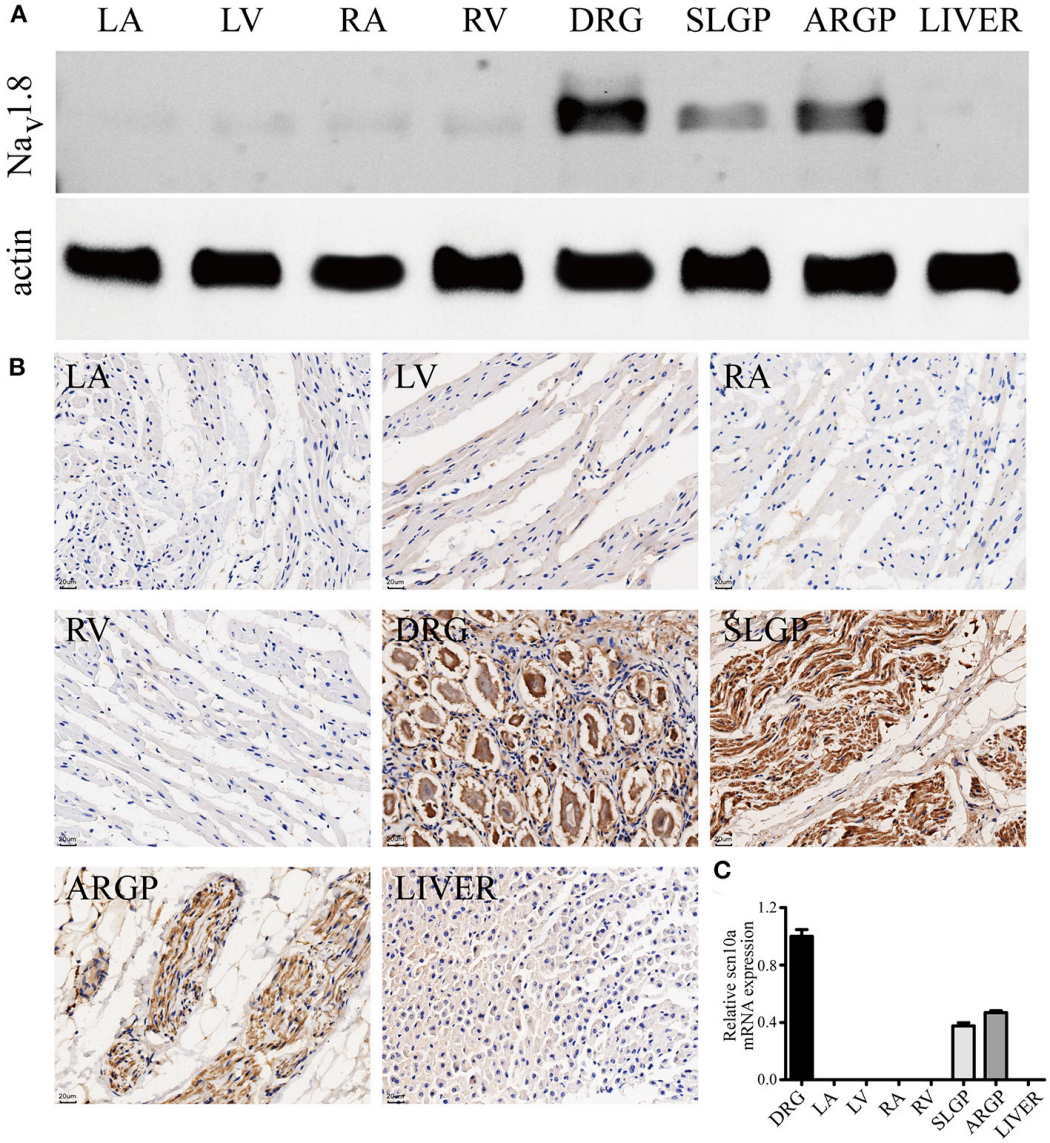
*Scn10a*/Na_V_1.8 expression in canine GPs and the myocardium. **(A)** Typical western blots for Na_V_1.8, *n* = 4. **(B)** Typical immunohistochemistry-stained sections for Na_V_1.8, scale bar = 20 μm, *n* = 4. The results confirmed the presence of Na_V_1.8 proteins in canine GPs but rarely expression in atria and ventricles. **(C)** *Scn10a* transcripts were readily detected in both superior left ganglionated plexi (SLGP) and anterior right ganglionated plexi (ARGP) but were not detected in right atrium (RA), left atrium (LA), left ventricle (LV), and right ventricle (RV) by qPCR. *n* = 4. Dorsal root ganglia (DRG) was used as the positive control and the liver was used as the negative control.

Na_V_1.8 protein expression in canine GPs and the myocardium was detected by western blot analysis and immunohistochemistry staining (Figures 5A,B). The results confirmed the presence of Na_V_1.8 proteins in canine GPs but rarely expression in atria and ventricles. We also examined the relative abundance of *scn10a* transcripts in canine GPs and the myocardium by qPCR (Figure 5C). *Scn10a* transcripts were readily detected in both SLGP and ARGP but were not detected in atria and ventricles.

### Size of the Ischemic Area

A well-defined borderline was present between the ischemic zone and the non-ischemic cardiac tissue. Table 1 summarized the left ventricle and septum myocardial mass of the ischemic area in the two groups. There was no significant difference between the two groups.

**Table 1:** Percentage of ischemic myocardial mass (%).

	**Septum**	**Left ventricle**
Control group (*n* = 10)	27.4 ± 2.5	30.3 ± 2.4
A-803467 group (*n* = 10)	25.7 ± 1.9	28.5 ± 2.2
*P*	>0.05	>0.05

## Discussion

The present study demonstrated for the first time that blocking Na_V_1.8 in cardiac GPs promotes occurrences of ventricular arrhythmias including VPCs, VT and spontaneous VF in the AMI model. Blockade of Na_V_1.8 shortened VERP, ventricular APD_90_, and decreased VFT during AMI. These effects may be mediated by inhibiting cardiac GP activity, as evidenced by the attenuation of the slowing of HR response to GP stimulation.

Of note, *SCN10A*/Na_V_1.8 was readily detected in canine GPs, but was not validated in canine ventricles by qPCR and western blot. Immunohistochemistry on canine tissue sections showed Na_V_1.8 labeling in cardiac GP (4), and A-803467 significantly reduced action potential firing frequency in GP neurons but did not affect cardio-myocyte action potential upstroke velocity (6). Low to absent expression levels of *scn10a* were observed in rabbit ventricular tissue, human atrial tissue and hiPSC-CMs (10). It has been reported that Na_V_1.8 is up-regulated in the human hypertrophied myocardium and the failing human myocardium, suggesting that inhibition of Na_V_1.8 could be an antiarrhythmic therapeutic target (14, 15). However, they made no attempt to identify the presence of Na_V_1.8 in intracardiac neurons or study their effects. We previously reported that blockade of Na_V_1.8 by A-803467 suppresses AF inducibility and cardiac conduction during vagus nerve stimulation, most likely through inhibiting GP activity (11). In order to localize the effects on cardiac GPs and minimize its systemic action on the myocardium, we intentionally injected A-803467 into cardiac GPs. In the present study, A-803467 can shorten APD_90_ and VERP, which accompanied by increasing incidences of VF and VT during myocardial ischemia. We have shown that inhibition of Na_V_1.8 channels increase the incidence of ventricular arrhythmias in AMI hearts through modulating GP activity.

The cardiac GP exerts a significant role in the initiation and maintenance of AF, and radiofrequency GP ablation is demonstrated to improve the success rate of AF ablation (16). The cardiac cholinergic neurons could also modulate ventricular electrophysiology. Pharmacological blockade or mechanical disruption of parasympathetic innervation decreased ventricular cAMP levels, shortened VERP, and increased the incidence of ventricular arrhythmias (17). He et al. reported that VF was significantly facilitated and ventricular arrhythmia incidence was significantly increased after GP ablation in AMI dogs (18). The present study also showed that suppression of GP activity may result in an imbalanced modulation of the heart, which may promote the genesis of ventricular arrhythmias during myocardial ischemia.

A study by Yu et al. showed that inhibition of Na_V_1.8 attenuated ischemia-induced ventricular arrhythmia by suppressing left stellate ganglion activity (19). At first glance, our finding may appear inconsistent with Yu's study; it is not. The cardiac GP contains entities representing both parasympathetic and sympathetic neurons, while a majority of GP neurons has been found to be parasympathetic (20). GP activation by HFS can evoke negative chronotropic effects and affect ventricular repolarization properties (18), and GP ablation can significantly decrease the ERP of the ventricular myocardium (21). Blocking Na_V_1.8 in left stellate ganglion could decrease sympathetic activity and attenuate ischemia-induced ventricular arrhythmia, however, blocking Na_V_1.8 in GP could inhibit parasympathetic activity and increase ischemia-induced ventricular arrhythmia.

The GP neurons could synthesize many different neurotransmitters. Acetylcholine is one of the principal excitatory neurotransmitters in the cardiac GP. Blasius et al. found that mice carrying the hypermorphic mutation of *SCN10A*, with enhanced Na_V_1.8 sodium currents, exhibited marked R-R variability and sinus bradycardia upon “scruffing,” which could be abrogated by atropine infusion (22). Brack et al. reported that nitric oxide can play a vagal protective role of suppressing VF, possibly through modulating APD restitution in rabbit hearts (23). We hypothesize that Na_V_1.8 could possibly modulate release of neurotransmitters in the cardiac GP, and nitric oxide or acetylcholine may represent potential candidates.

Our study has some limitations. First, we did not record neural activity directly within the GP. However, we have provided the evidence of altered GP function which could correlate well with GP neural activity based on prior studies (12). Second, other potentially important GPs were not studied, such as the GP near the ligament of Marshall. However, a complete GP blockade might be difficult to achieve. Third, the signaling pathway mediating the protective role of Na_V_1.8 remains to be determined. Our further study will be performed to clarify the exact mechanism and the downstream pathways involved in the antiarrhythmic actions of Na_V_1.8.

In view of the present results, as suppression of GP activity may promote the genesis of ventricular arrhythmias, GP ablation should be avoided in patients with ischemia diseases. Our results may identify Na_V_1.8 as a potential novel therapeutic target for antiarrhythmic intervention aiming at modulating the neural control of the ischemic heart to treat patients with refractory ventricular arrhythmias or electrical storm. Our study may lead to the development of a novel oral Na_V_1.8 stimulator with more specific action and less adverse effects.

In conclusion, *SCN10A*/Na_V_1.8 is detectible in canine GPs but not in ventricles, and blockade of Na_V_1.8 in cardiac GPs increases the incidence of ventricular arrhythmias in AMI hearts. Our study shows for the first time an influence of *SCN10A*/Na_V_1.8 on the regulation of ventricular arrhythmogenesis via modulating cardiac GP activity in the AMI model.

## Data Availability Statement

The original contributions presented in the study are included in the article/supplementary material, further inquiries can be directed to the corresponding author/s.

## Ethics Statement

The animal study was reviewed and approved by Institutional Animal Care and Use Committee of Zhongshan Hospital, Fudan University.

## Author Contributions

BQ, SD, YS, LC, and JG: conception, design, data analysis, and interpretation. BQ, LC, YS, DS, FL, and LW: administrative support. BQ, SD, CZ, ZN, and JL: collection and assembly of data. BQ: manuscript writing. All authors wrote first draft, read, amended the draft, and final approval of manuscript.

## Conflict of Interest

The authors declare that the research was conducted in the absence of any commercial or financial relationships that could be construed as a potential conflict of interest.

## Publisher's Note

All claims expressed in this article are solely those of the authors and do not necessarily represent those of their affiliated organizations, or those of the publisher, the editors and the reviewers. Any product that may be evaluated in this article, or claim that may be made by its manufacturer, is not guaranteed or endorsed by the publisher.
